# New treatments for influenza

**DOI:** 10.1186/1741-7015-10-104

**Published:** 2012-09-13

**Authors:** Sailen Barik

**Affiliations:** 1 Center for Gene Regulation in Health and Disease, Cleveland State University, 2351 Euclid Avenue, Cleveland, Ohio 44115, USA; 2 Department of Biological, Geological and Environmental Sciences, Cleveland State University, 2121 Euclid Avenue, Cleveland, Ohio 44115, USA

**Keywords:** Cathelicidin, defensin, influenza, hemagglutinin, neuraminidase, NSAID, oseltamivir, siRNA, zanamivir

## Abstract

Influenza has a long history of causing morbidity and mortality in the human population through routine seasonal spread and global pandemics. The high mutation rate of the RNA genome of the influenza virus, combined with assortment of its multiple genomic segments, promote antigenic diversity and new subtypes, allowing the virus to evade vaccines and become resistant to antiviral drugs. There is thus a continuing need for new anti-influenza therapy using novel targets and creative strategies. In this review, we summarize prospective future therapeutic regimens based on recent molecular and genomic discoveries.

## Review

### Background

Influenza, commonly known as 'flu', is a respiratory infection contracted by 5% to 50% of the US population annually, roughly 200,000 of whom are hospitalized and 25,000 die (with significant year-to-year variation) [1-4]. Clinically, influenza presents itself with high fever, chills, sore throat, headache, runny or stuffy nose, weakness, muscle pain and sometimes diarrhea (vomiting in children). Although more severe than common cold, influenza is generally a self-limiting disease in healthy adults that lasts about a week, but cough and lethargy may continue for some time. In the population, influenza follows the general pattern that now appears to characterize essentially all respiratory infections, in that it can be particularly hazardous to individuals with poor immunity such as children and the elderly, and those with pulmonary, cardiovascular or other complications. Pneumonia, either a direct result of the virus infection in the lung, or through secondary bacterial infections shortly after the viral episode, is also common in influenza, particularly among adults [2]. Secondary bacterial pneumonia often complicates influenza and in fact played a significant role in the morbidity and mortality associated with all past pandemics, including the most recent 'swine flu' of 2009 [2,5]. Prompt antibiotic treatment is required to reduce mortality. Relatively rare complications of influenza include myositis (muscle inflammation), myocarditis and pericarditis (affecting the heart), Reye's syndrome and possibly Guillain-Barré syndrome. Although the primary target and clinically relevant tissue in influenza virus infection is the respiratory epithelium [2], facultative infection of other organs, such as the cardiac or skeletal muscle, is possible and has occasionally been documented in cell culture and experimental animal infections [6-10]. The predominant mode of natural transmission of the influenza virus is by aerosols, generated by coughing or sneezing; however, it is also transmitted by nasal secretions and contact with contaminated surfaces. While all respiratory viruses, including influenza, use the nose as the common entry channel, they can also enter through the eye, likely via the tear duct, draining into the sinus and the airways [11]. The virus particles are inactivated by the ultraviolet rays in sunlight and common disinfectants such as soap. Thus, frequent hand washing is recommended during influenza epidemics to minimize virus spread.

**Figure 1:**
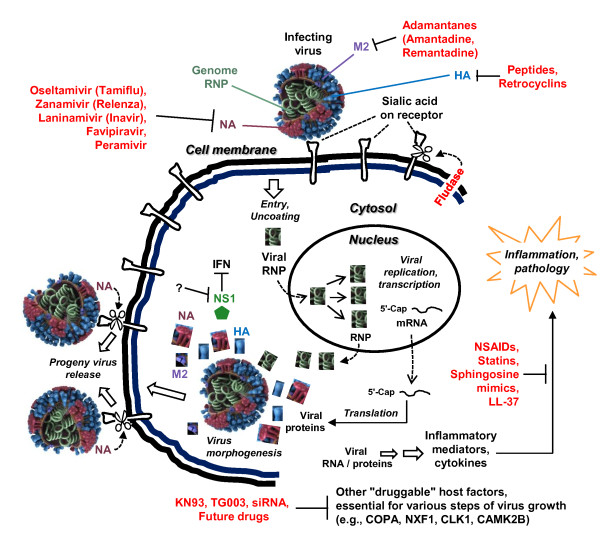
**Anti-influenza drugs and their biological targets**. The relevant viral proteins (color-coded) and old and new drugs targeting them are shown (not drawn to scale). The genomic ribonucleoprotein complex is shown as tightly coiled. Influenza viral RNA synthesis occurs in the infected host nucleus using this ribonucleoprotein as a template, while translation occurs in the cytoplasm. Neuraminidase (NA) and the drug candidate, Fludase, cleave the sialic acid receptor on the cell membrane, as indicated by the cutting scissors. Nonstructural proteins (only NS1 is shown) are not packaged in mature virions. Diverse viral products activate an inflammatory response that can be quelled by the use of anti-inflammatory treatments, such as non-steroidal anti-inflammatory drugs. Potential future drug regimens, targeting influenza-relevant cellular functions, are shown at the bottom. (Influenza virion image credit: Dan Higgins and Doug Jordan, CDC Public Health Photo Library, image #11822). HA: hemagglutinin; IFN: interferon; NA: neuraminidase; NS: nonstructural protein; RNP: ribonucleoprotein.

The influenza viruses are RNA viruses of the *Orthomyxoviridae *family, in which the viral genome is divided into multiple segments [4]. For example, the total genome of influenza A, which is responsible for the vast majority of seasonal influenza in humans, consists of eight negative sense (anti-mRNA sense) RNA segments. Together, they code for 10 viral proteins: three subunits of viral RNA-dependent RNA polymerase (RdRP) (PA, PB1, PB2); major surface glycoproteins, hemagglutinin (HA) and neuraminidase (NA); nucleocapsid protein (NP); matrix proteins (M1, M2); and two nonstructural proteins, NS1 and NS2 [4]. In some strains of animal influenza virus, the PB1 gene also produces a small, 87-residue protein, named PB1-F2, by internal translational initiation of an alternate reading frame; this protein shows a predominantly mitochondrial localization and promotes apoptosis in immune cells, likely aiding viral transmission [12]. The influenza viral genomic RNA is wrapped with the NP protein and the resultant ribonucleoprotein (NP-RNA) is transcribed by the viral RdRP to produce viral mRNAs that serve as templates for viral protein synthesis. The NP-RNA complex is encapsidated in a lipid bilayer, studded with the HA and NA glycoproteins and traversed by the M2 protein (Figure 1), which is an ion (proton) channel [4]. The nonstructural proteins are so named because they are not packaged into mature virus particles; however, they play essential roles in the infected cell. NS1 interacts with a large number of host proteins including several members of the innate immune pathways [13-20], and hence contributes to virus growth, pathogenicity and tropism [21-24]. NS2, also called nuclear export protein, mediates nucleus-to-cytoplasmic export of the viral RNA by acting as an adaptor between viral ribonucleoprotein complexes and the nuclear export machinery of the cell.

Influenza viruses are divided into subtypes A, B and C, based on genetic and antigenic differences in their HA and NA surface glycoproteins [4,25]. Seasonal human influenza is caused by both types A and B, whereas C is rare and only causes a mild disease in children [3,4]. The type A viruses also naturally infect a variety of nonhuman species, including birds, pigs, horses, cats, dogs, seals and whales [3,25-28]. There are 16 known HA (H1 to H16) and 9 NA (N1 to N9) subtypes in influenza A [4,25], leading to the current HxNy nomenclature. Routine human infections of seasonal influenza are mainly due to H1N1, H1N2 and influenza B; however, H3N2 is gradually becoming more abundant [29]. In 2011, a new variant of H3N2, sometimes referred to as H3N2v, was found in a dozen patients in the US. The more deadly pandemics and epidemics have been caused by various mutant variants and subtype combinations. The 1918 'Spanish flu' and the 2009 'swine flu' were both caused by H1N1 type viruses, the 1957 'Asian flu' was caused by an H2N2, the 1968 'Hong Kong flu' by H3N2 and the 2004 'bird flu' by H5N1. Antigenic drift within a specific HA or NA number is also common (see 'Difficulties of prevention and treatment of influenza' below).

An interesting and clinically relevant aspect of pandemic and epidemic influenza that sets it apart from seasonal influenza is the induction of the so-called cytokine storm, consisting of interleukin-6, tumor necrosis factor α and interferon-γ. Together, these proinflammatory cytokines cause systemic inflammatory response syndrome, leading to multiorgan failure that includes airways destruction, vascular endothelial damage and plasma leakage [30-35].

### Difficulties of prevention and treatment of influenza

There are a number of difficulties in influenza treatment and prevention, contributing to the constant threat of the disease. These are summarized below. A prior understanding of these factors is clearly important in strategizing new treatments.

#### Rapid mutability

Like all RNA genomes, the influenza virus genome lacks a proofreading mechanism and thus mutates relatively frequently. Mutations may offer the virus various selective advantages, such as resistance to existing vaccines and antiviral drugs [36] - even small changes in the viral HA and NA antigen sequences, known as antigenic drift, may allow the virus to escape from the host's adaptive immunity [37]; increased infectivity and virulence; and greater horizontal spread (that is, one individual to another in the same species) and vertical spread (that is, crossing of the host species, such as from pig to man, generally due to a 'variant' virus). Larger diversity and more extensive changes can be rapidly generated by genetic reassortment, as described below.

#### Genomic reassortment

As the influenza genome is segmented (multiple pieces), new strains can quickly appear by reassortment in co-infection. Known as antigenic shift, this often leads to hybrid strains that are markedly different [25]. For example, co-infection by human and swine (pig) influenza viruses can generate reassortant viruses that will have genomic RNA segments from the two viral species [38]. Such a hybrid can cause a major epidemic because the human population will lack any natural immunity to it. It has been speculated that the 1918 influenza virus, which caused the largest influenza pandemic recorded in history, was caused by such a reassortant virus [39,40]. The 2009 Mexican swine influenza is likely a product of multiple assortments between swine, human and European avian-like strains [41,42]. Obviously, reassortment may result in a new combination of the HA and NA segments, thus changing the subtype name of virus as well.

#### Vulnerable population groups

Influenza can be particular deadly to specific groups in the population, such as the elderly and individuals with diabetes or immune deficiency (such as those with AIDS). In fact, people aged 65 years or older account for 90% of seasonal influenza-associated deaths, even though this group makes up only approximately 15% of the population [1,2]. Thus, this group is in the greatest need of prophylaxis or more intensive treatments against influenza but, unfortunately, they are also generally less tolerant to aggressive treatments.

The brief background presented above should make it clear that reliable prevention and treatment of influenza is a critical need in public health. In this review, we start by summarizing the various current and now-defunct treatments for influenza (amantadine, oseltamivir (Tamiflu), zanamivir (Relenza)), as there are lessons to learn from their success and failure. We then discuss and critically review the prospective future anti-influenza treatments that are at different stages of development (newer NA inhibitors, sialidase, defensins, cathelicidin, statins, siRNA and host proteins).

### Current and past treatments

#### M2 ion channel inhibitors: adamantanes

The influenza viral M2 protein acts as an ion channel that allows proton translocation through the virion envelope (Figure 1). This leads to acidification of the viral core, its resultant dissociation and the release of the viral NP-RNA complex in the infected cell cytoplasm, which is essential for viral RdRP function and viral gene expression [4]. The M2 inhibitors are adamantanes, characterized by three condensed cyclohexane rings fused in the chair conformation. Two M2 inhibitors, amantadine and rimantadine, were widely used against influenza but are now largely discontinued and replaced by NA inhibitors [43]. As one would predict from the role of M2 in the viral life cycle, these drugs were effective only when administered soon after diagnosis [43]. Nonetheless, their efficacy is limited to influenza A only, since influenza B viruses lack M2. Moreover, essentially all influenza strains have now developed high resistance against both amantadine and rimantadine. This has been attributed to their easy over-the-counter availability in highly populated nations such as China and Russia, and their large-scale use in the poultry for protection against 'chicken flu'.

### Old neuraminidase inhibitors

#### Oseltamivir (Tamiflu)

**Figure 2:**
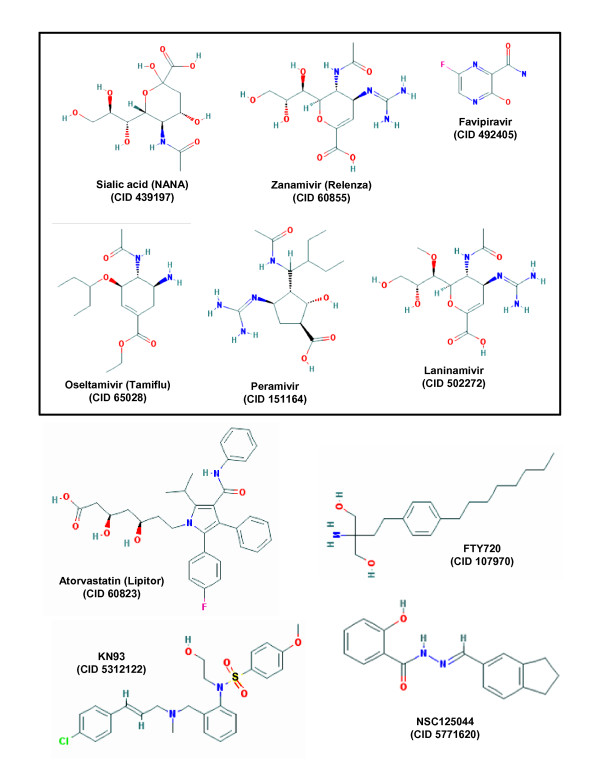
**Structure of selected class-representative anti-influenza drugs, old as well as prospective ones**. The neuraminidase inhibitors, and N-acetyl neuraminic acid that they mimic, are shown in the box. For all molecules, the PubChem compound numbers (CID#) are written under each name. In FTY720, one of the two -OH groups is phosphorylated to yield the bioactive phosphate derivative (not shown). The structure of AAL-4 is similar (not shown), but it has only one -OH group instead of two, which is phosphorylated much faster. M2 inhibitors are not shown for lack of space and because they are largely discontinued due to viral resistance. All structures were obtained from the free PubChem Compound Database at National Center for Biotechnology Information (accessed June 15, 2012) [44]. NANA: N-acetyl neuraminic acid.

The most popular influenza treatment regimen, developed nearly three decades after the M2 inhibitors, targets the viral NA. The NAs possess glycoside hydrolase activity that cleaves the glycosidic linkages of neuraminic acids. The influenza virus uses viral HA, a virion surface protein, to bind to sialic acid groups (Figure 2) on cell surface glycoproteins (Figure 1) [45]. For the progeny virions to be released from the cell, the NA activity must cleave the sialic acid groups from the host glycoproteins, and this is essential for viral spread and reinfection (Figure 1). Thus, blocking the function of NA with specific inhibitors is an effective way to treat influenza.

The influenza NA is a classic example of rational drug design based on the crystal structure of NA [46,47]. Currently, two NA inhibitors are used in clinical practice: oseltamivir (Tamiflu; Roche/Genentech) and zanamivir (Relenza; GlaxoSmithKline) (Figure 2). The 2009 H1N1 pandemic witnessed record sales of both drugs, together exceeding US $4 billion, partly due to stockpiles for fear of a forthcoming epidemic. Both drugs bind the catalytic pocket of NA and function as competitive inhibitors of NA activity.

Oseltamivir as a prodrug is sold in capsules containing 30 mg, 45 mg or 75 mg oseltamivir phosphate and also as powder for oral suspension in water (6 mg/mL). For treatment of influenza, the recommended dose for adults is 75 mg, twice a day, for 5 days. The preventive (prophylactic) dose is usually 75 mg, once a day for at least 10 days, or for up to 6 weeks during a community influenza outbreak. Smaller doses are recommended for children, according to age and weight. Adverse drug reactions may include nausea, vomiting, diarrhea, abdominal pain, headache and neuropsychiatric events such as self-inflicted injury and delirium (Table 1) [36]. As with many drugs, such as the M2 inhibitors [43], oseltamivir may be less effective if used in late-stage influenza.

**Table 1:** Old and new influenza drugs

Name (major brand)	Effective against	Recommended dose	Use status; adverse drug reactions
**Amantadine **[43] **(Symadine, Symmetrel) **[43]	Influenza A	Capsule/tablet, syrup; 100 mg amantadine hydrochloride, twice a day.	Mostly discontinued due to resistance; may be recalled in future epidemics.
**Oseltamivir (Tamiflu) **[48,49]	Influenza A, B	Capsule (30, 45, 75 mg) twice a day; powder for suspension (6 mg/mL).	Currently in use. Transient nausea, vomiting, abdominal pain, headache, neuropsychiatric episodes.
**Zanamivir (Relenza) **[36]	Influenza A, B	Two inhalations (5 or 10 mg each).	Currently in use. Relatively rare adverse drug reactions include nausea, diarrhea, respiratory problems, dizziness.
**Laninamivir **[50,51]	Influenza A, B (for example, H1N1, H3N2)	Single inhalation (20 or 40 mg).	Similar to oseltamivir. Approved in Japan, but not yet in the US.
**Peramivir **[52]	Similar to Laninamivir	Intravenous 600 mg once, or 300 mg twice, 5 to 10 days.	Transient nausea, vomiting, and diarrhea (similar to oseltamivir). Approved in Japan and Korea.

Some information was obtained from manufacturers' inserts and/or websites.

#### Zanamivir (Relenza)

Zanamivir is more effective than oseltamivir and is supplied for oral inhalation only [36]. It is sold as double-foiled 'blisters' that release the drug in an inhaler when pierced by the user. Each blister contains a powder mixture of 5 mg of zanamivir and 20 mg of lactose (plus milk proteins). The recommended dose for treatment of influenza in adults and pediatric patients aged 7 years and older is 10 mg twice daily (that is, two inhalations, one 5-mg blister per inhalation) for 5 days. For prevention of influenza (prophylaxis), the recommended dose in adults and pediatric patients 5 years and older is 10 mg once daily for 10 days, inhaled as above. Adverse drug reactions are rarer than with oseltamivir (Table 1).

#### Resistance to oseltamivir and zanamivir: what they tell us

Influenza virus mutants, resistant to either drug, have been characterized from cell culture as well as from patients [43,48,49] and are particularly well-studied for oseltamivir. Interestingly, resistant mutations were found not only in the NA gene, but also in HA. It appeared that, at least in cell culture, two HA or an HA and NA mutation can act synergistically to increase resistance [53]. As expected, the NA mutations were in conserved catalytic (Arg152, Arg292) and structural (Glu119, Asp198, His274, Asn294) residues. A few common mutations were: R152K, R292K, E119V, D198N, H247Y (highly prevalent) and N294S [54]. Double mutations with synergistic oseltamivir resistance phenotype have been noted as well. This includes the E119V+I222V double mutant, isolated from an immunocompromised child infected with H3N2 virus, and H247Y+I222V, from patients infected with the H1N1 virus of the 2009 pandemic.

For future drug design and resistance expectation, it is important to learn that the effect of these mutations is dependent both on the NA subtype and the drug used [55,56]. Generally, catalytic site mutants exhibit drug resistance, but also show decreased NA activity, such that viral infectivity, pathogenicity and transmissibility are affected. By contrast, mutations of the structural residues exhibit drug resistance without a significant effect on NA functionality. Thus, in natural selection against these drugs, the structural mutations may be favored because they would retain viral fitness. Lastly, the HA mutations tend to map to regions associated with receptor binding of the HA, apparently lowering the affinity of the HA for the cellular receptor, such that NA is no longer required for virus release.

Readers interested in the detailed dosage of the existing drugs (amantadine, rimantadine, oseltamivir and zanamivir) are encouraged to read the highly comprehensive treatise of the Advisory Committee on Immunization Practices from the National Center for Immunization and Respiratory Diseases, Center for Disease Control and Prevention, USA [57].

### New and prospective future treatments

#### New and future neuraminidase inhibitors

##### Laninamivir

Recently, a new NA inhibitor, laninamivir (Inavir; Daiichi-Sankyo and Biota; Figure 2), has been approved for use in Japan [50,51], and is currently being developed in the US. It is a highly promising and long-acting NA inhibitor that efficiently inhibits common oseltamivir-resistant viruses, including those with the H274Y substitution [58]. Co-crystal structure of laninamivir-NA has revealed that laninamivir-binding shares some of the same residues as oseltamivir and zanamivir [58]. Nonetheless, the three drugs differ in their pharmacokinetics [52]. Laninamivir is only available for inhalation, and a single inhalation has been shown to be as effective as repeated doses of oseltamivir or zanamivir [50], likely due to its long persistence in the lung. The single use regimen is expected to promote improved patient compliance and convenience [51].

##### Favipiravir

The second new investigational drug against NA is T-705 (favipiravir; 6-fluoro-3-hydroxy-2-pyrazinecarboxamide; Figure 2) [59] that has shown antiviral activity against seasonal influenza viruses as well as oseltamivir-sensitive or -resistant highly pathogenic H5N1 viruses [60]. Moreover, its active form is a ribofuranosyl triphosphate derivative that mimics purines or purine nucleosides and inhibits the viral RdRP but does not inhibit human polymerases [59]. Thus, favipiravir shows excellent promise for the treatment of patients with the highly pathogenic H5N1 influenza. The National Institutes of Health of the US is currently conducting a Phase II, randomized, double-blind, placebo-controlled, multicenter (in 235 study locations) study evaluating the efficacy and safety of favipiravir in adult patients with uncomplicated influenza (ClinicalTrials.gov identifier NCT01068912; sponsor: FujiFilm Pharmaceuticals USA, Inc.). A 5-day regimen is being tested with low-dose (1000 mg favipiravir twice for 1 day, followed by 400 mg favipiravir twice a day for 4 days) as well as high-dose favipiravir (1200 mg favipiravir twice for 1 day, followed by 800 mg favipiravir twice a day for 4 days). The results are expected to be available in late 2012 or early 2013.

##### Peramivir

The third new compound in the NA-inhibitor category is peramivir (Biocryst Pharmaceuticals; Figure 2). It is the only intravenous (IV) option used for the treatment of certain hospitalized patients with known or suspected 2009 pandemic H1N1 influenza, but its approval by the US Food and Drug Administration expired soon after the pandemic. A phase III clinical trial of parenteral (IV) peramivir, conducted on 230 patients in 110 study locations and sponsored by the US Department of Health and Human Services, was recently completed (ClinicalTrials.gov identifier NCT00957996; sponsor: BioCryst Pharmaceuticals). It tested the safety and tolerability of peramivir administered either as a once-daily infusion of 600 mg or a twice-daily infusion of 300 mg to adult and adolescent patients hospitalized with confirmed or suspected influenza infection. Both dose regimens of IV peramivir were found to be safe and well-tolerated. Another phase III study of IV peramivir has also been initiated and continues at the time of this writing (ClinicalTrials.gov identifier NCT00958776; sponsor: BioCryst Pharmaceuticals). Because of its intravenous applicability, peramivir is particularly useful when a patient has developed resistance to oseltamivir and is unable to inhale zanamivir (for example, patients with asthma), the two major anti-influenza drugs. Peramivir is already being sold in Japan under the trade name Rapiacta, and in South Korea under the name Peramiflu.

The promise of such new generation NA inhibitors suggests that NA may continue to provide a rational target for newer inhibitors in the future, effective against viruses that will develop resistance to the older inhibitors [46].

### Hemagglutinin inhibitors

#### EB peptide

In an interesting report [61], a 20-amino-acid peptide (RRKKAAVALLPAVLLALLAP), derived from the signal sequence of fibroblast growth factor 4, specifically bound to the influenza viral HA protein (Figure 1) and exhibited broad-spectrum antiviral activity against influenza viruses including H5N1. Named EB for Entry Blocker, the peptide was also protective when administered post-infection, suggesting that it prevented reinfection, which underscored its therapeutic potential.

#### Peptide NDFRSKT

In a complementary approach [62], a heptapeptide phage display library was biopanned against purified avian influenza virions of subtype H9N2. Multiple rounds of panning and antiviral screening led to the identification of the peptide NDFRSKT with strong antiviral properties. The peptide inhibited the hemagglutination activity of the viruses but not the NA and hemolytic activities. Further studies confirmed that the peptide directly interacted with the HA protein. The therapeutic status of the peptide remains unknown.

### Fludase, a neuraminidase mimic

Fludase (DAS181) is a recombinant chimeric enzyme in which a fungal sialidase catalytic domain is fused to a cell surface-anchoring domain [63,64]. Enzymatically, it functions essentially like the viral NA and destroys the host cell surface sialic acid receptors of the virus (Figure 1). Thus, it differs from the NA inhibitors in two respects: it is a protein, not a small compound; and it targets the host cell rather than the virus itself. In preclinical studies, Fludase inhibited both human and avian lethal influenza viruses [63,64]. Fludase is designed and developed by NexBio (http://www.drugdevelopment-technology.com/projects/fludase); however, its future development by the company remains uncertain.

### Anti-inflammatory drugs

With the recognition that the body's hyperactive inflammatory response is a root cause of organismic and systemic damage in many pathological states, efforts at quelling inflammation have received pharmaceutical attention [65-67]. Notable direct and indirect anti-inflammatory regimens, tested in various infections, include corticosteroids, aspirin (a common non-steroidal anti-inflammatory drug), monoclonal antibodies, antagonists of cytokines and chemokines, statins and sphingosine analogs. They might be particularly helpful in pandemic events, which, as mentioned before, are characterized by exaggerated synthesis of proinflammatory cytokines, known as a cytokine storm [68]. Although anti-inflammatory drugs have produced mixed, and sometimes conflicting, results in patients with influenza, a few are worth mentioning here.

#### Statins

Statins inhibit cellular 3-hydroxy-3-methylglutaryl-coenzyme A (HMG-CoA reductase), an enzyme essential for cholesterol biosynthesis in the liver, and are extensively prescribed to treat hypercholesterolemia [69]. Statins are relatively safe with rare incidents of myositis, myopathy and neuropathy. Thanks to the generally accepted correlation of high serum cholesterol levels and predisposition to atherosclerosis, multiple statins are currently blockbuster pharmaceuticals that include: atorvastatin (Figure 2) (Lipitor by Pfizer), lovastatin (Mevacor by Merck & Co.), and simvastatin (Zocor by Merck & Co.), as well as generic varieties.

In relatively recent approaches, statins have been tested in influenza, based on the premise that they might reduce the mortality and morbidity caused by the cytokine storm [30,65,66,70,71]. However, these studies have generated contradictory claims. In one retrospective study [72], a database of 3,043 adults in the US hospitalized with laboratory-confirmed influenza during the 2007 to 2008 influenza season was analyzed. Of these patients, 1,013 received statins and 151 died within 30 days of their influenza test. The analysis revealed a positive correlation of statin use with reduced mortality. By contrast, when 1,520 patients in the UK [73] with confirmed 2009 pandemic influenza A (H1N1) infection were surveyed for pre-admission statin use and in-hospital severity, no significant correlation could be found. Another recent study in Spain [74] examined the use of corticosteroids, macrolides and statins among 197 patients with 2009 pandemic influenza H1N1, who also had complications from pneumonia, suggesting a role of the inflammatory response. Unfortunately, none of these immunomodulatory therapies was found to be associated with a lower risk for developing severe disease. The apparent variability among some of these studies may be due to a number of factors [75], such as subtle differences in the viral genome sequence between the two pandemics, the dose and frequency of statin use, and environmental factors. Clearly, a more detailed, focused and controlled clinical trial is needed to evaluate the benefit of statin use, perhaps in conjunction with an antiviral agent such as oseltamivir or zanamivir.

#### Sphingosine mimics

Sphingolipids are a family of lipid mediators, of which sphingosine and its phosphate (sphingosine 1-phosphate or S1P) have been recognized as modulators of diverse cellular activities. The sphingosine analog family is a group of recent immunosuppressants with high therapeutic potential for influenza. In the body, these compounds mimic natural sphingosine and are first phosphorylated by sphingosine kinase [76,77]. The phosphoform then acts as an agonist of multiple types of sphingosine receptors, which leads to lymphopenia through the sequestration of lymphocytes in the lymph nodes, resulting in immunosuppression [76,77]. Fingolimod (FTY720) (Figure 2), an early member of this family (trade name Gilenya, from Novartis), is derived from a fungal metabolite and currently approved for treatment of autoimmune conditions, multiple sclerosis, cardiac failure and arrhythmia. Later, removal of a hydroxyl group in FTY720 generated AAL-4, which was much more rapidly phosphorylated in humans, improving its efficiency [78]. A series of studies have now shown that AAL-4 provides significant protection against the cytokine storm in pathogenic influenza by limiting pulmonary injury [79,80]. In a representative study [80], mice were intranasally infected with the pathogenic pandemic A/Wisconsin/WSLH34939/09 influenza virus and an hour later treated with AAL-R (0.2 mg/kg in 100 µL water intratracheally) or 100 µL water alone. These animals with compared with those receiving 5 mg/kg (also in water) of oseltamivir (Tamiflu) by gavage. The results showed that AAL-R administration alone significantly lengthened the survival time of the animals (82%) compared with those that received water. As expected, oseltamivir treatment alone significantly increased the number of survivors (50%) compared with just water (21%); however, protection was significantly less than that from AAL-R treatment (50% versus 82%). Interestingly, a combination of AAL-R and oseltamivir resulted in 96% survival, which is greater than either drug alone. Hence, a dual drug cocktail of a direct viral function inhibitor and a host immune response inhibitor may be a promising approach in the treatment of influenza.

#### Nuclear factor-kappaB inhibitors

NF-κB is a transcription factor of many genes of the cellular innate immune pathway and its activation underlies a variety of antiviral as well as inflammatory responses that range from septic shock to cancer. In fact, NF-κB has been considered a major target of immunomodulatory and anti-inflammatory therapy [81,82]. In its interesting dual role, NF-κB is not only a critical contributor of cytokines and interferon synthesis in influenza infection, but also essential for the growth of the virus itself [83-87]. Although the exact mechanism for the latter remains unclear, the balance must be tightly regulated, as the influenza viral NS1 protein actually inhibits NF-κB [88]. Clearly, even after the inhibition, enough active NF-κB persists to promote viral growth and the inflammatory response. Thus, inhibitors of NF-κB may have a two-pronged beneficial effect in influenza: they will inhibit the virus directly and will also moderate the systemic inflammation. This has been shown in cell culture and mice [84-86], but specific and controlled studies in patients with influenza still need to be done. Nonetheless, the prospects of an anti-inflammatory therapy of influenza are real, because some anti-NF-κB drugs, such as acetyl salicylate (aspirin) are routinely sold in stores without prescription and widely used by the general public for many years without major side effects.

### Antimicrobial peptides and proteins

**Figure 3:**
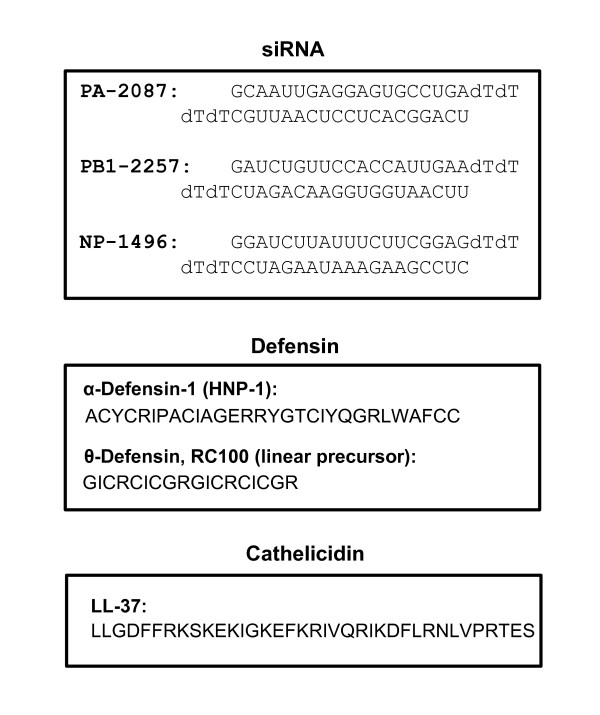
**Sequences of selected anti-influenza macromolecules**. Three representative classes are shown (siRNA, defensin and cathelicidin). Experimentally successful siRNA against influenza PA, PB1 and NP genes are shown in the upper box [90]. For each siRNA, the location of the sequence in the original gene is indicated by nucleotide number; thus, PA-2087 indicates an siRNA in which the first nucleotide at position 2087 of the PA gene. The upper strand is written 5′to 3′; the two deoxythymidine (dT) at the 3′-end are presumed to stabilize the siRNA [91]. The lower box shows the 37-mer peptide LL-37, written in single letter codes [92-94]. In the defensin family, note the abundance of Arg and Cys residues that are important for function [89].

A variety of animal and plant species produce small antimicrobial peptides and larger proteins that exhibit innate immune functions against an increasing number of pathogens. The two major families of antimicrobial peptides are defensins and cathelicidins (Figure 3) [89], whereas the collectins are larger proteins. Although antimicrobial peptides are diverse, they are generally cationic and amphipathic, which allows them to interact with and disrupt microbial membranes. In addition, they modulate the immune system by inducing the production of proinflammatory cytokines, act as chemokines for neutrophils and enhance phagocytosis of macrophages [89]. Recent studies have revealed antiviral - including anti-influenza - activities of some of these molecules, some of which are presented here.

#### Defensins

The defensins typically contain six Cys residues, forming three intramolecular disulfide bonds that regulate their structure and function (Figure 3) [89]. They are subdivided into α-, β- and θ-defensins, depending on their molecular weight and location of the Cys-Cys bonds. A number of defensins inhibited influenza virus growth, but the mechanism remains a matter of debate.

Humans have six α-defensins, of which α-defensin-1 (also known as human neutrophil peptide-1, HNP-1) and α-defensin-2 (HNP-2) were shown to increase neutrophil uptake of influenza virus [95,96]. The β-defensins had a significantly lower activity in this particular mechanism. In another study, HNP-1 (Figure 3) exhibited anti-influenza activity in epithelial cell culture as well, inhibiting viral RNA and protein synthesis [97]. Pretreatment of the cells with HNP-1 also inhibited viral replication, showing that the inhibition was due to modulation of cellular pathways. Protein kinase C was shown to be inhibited by HNP-1 treatment, suggesting the involvement of the protein kinase C pathway in nonimmune cells.

The θ-defensins, also called retrocyclins, are uniquely circular 18-residue peptides formed by post-translational joining of the N- and C-termini of two nonapeptides, and they also occur exclusively in primates [89,96]. The human θ-defensin genes are intriguing exceptions as they are pseudogenes harboring mutations that prevent the production of θ-defensin proteins. Studies of nonhuman primate θ-defensins have revealed that they are lectins with glycoprotein-binding properties that can inhibit fusion of HIV with the host cell, thus suggesting a novel antiviral regimen [98-101]. Human β-defensin 3, another lectin, also inhibited HA-mediated influenza viral fusion in a similar way [101]. Recently, retrocyclin-1 (Figure 3) and its various synthetic analogs, some with structural variations (for example, hapivirins, diprovirins) were tested against influenza virus in human cell culture and shown to block infection at low micromolar concentrations [99,101,102]. The success of synthetic peptides has opened the possibility that further engineering of the defensin sequences may lead to more optimized anti-influenza efficacy and pharmacological properties [100,103]. In an interesting complementary approach, aminoglycosides (amikacin 40 µg/mL; gentamicin 5 µg/mL; tobramycin 10 µg/mL), which are known to promote suppression of termination by codon-misreading [104], were used to produce retrocyclin from the endogenous human genes, and this also resulted in resistance to HIV-1 [103]. Although aminoglycosides are sometimes prescribed to fight serious bacterial infections, nephrotoxicity and ototoxicity are relatively frequent [105]. It remains to be seen whether an optimized dose will generate enough retrocyclin to block influenza viral fusion without causing unacceptable toxic effects in the patient with influenza.

A few defensin mimetics are currently being developed by PolyMedix (Radnor, PA, USA) and are at various stages of preclinical and clinical trials, but apparently none is being tested against influenza.

#### Cathelicidins: LL-37

In humans, the cationic antimicrobial protein hCAP18 is cleaved between Ala103 and Leu104 to generate LL-37, a 37-residue peptide with two tandem Leu residues at the amino terminus (Figure 3). Recent studies are revealing these peptides are not just antibacterial molecules but have a variety of innate immune functions [92,93]. LL-37, which is expressed in a number of cell types including epithelial, was recently shown to protect mice against influenza [94]. Using a lethal dose of the two different influenza A strains (A/PR/8/34 H1N1 and A/Udorn/307/72 H3N2), significantly higher survival and decreased weight loss was observed in LL-37-treated animals, which compared favorably with the Relenza-treated positive controls. Although a part of the better prognosis could be due to suppression of the inflammatory response by LL-37, the accompanying lower pulmonary viral titer, the short window of the acute infection, and the reproducibility of the viral inhibition in cell culture all point to a direct antiviral role of LL-37, the mechanism of which remains to be determined [94]. The LL-37 in these mouse experiments was administered with a respiratory nebulizer at a concentration of 500 µg per milliliter of saline and compared with the same concentration of Relenza [94]. At least in cell culture, the virus-inhibitory concentration of LL-37 approximated its natural concentration in the human lung [94]. LL-37, promising as it is, awaits further development for influenza treatment. It also remains to be seen whether other such antimicrobial peptides have antiviral, and specifically anti-influenza, properties. If so, this family may constitute an exciting and novel regimen for influenza treatment in the future.

#### Collectins

The collectins belong to the superfamily of collagen-containing C-type lectins (hence their name), and act as pattern recognition receptors for pathogenic molecules [106,107]. Better known members include the mannan-binding lectin and the surfactant proteins A and D. There is a large body of literature documenting an innate antiviral role of these lectins, in which they neutralize viral infectivity by binding to viral fusion glycoproteins, such as influenza viral HA and NA proteins; however, their contributions in opsonizing viral antigens and triggering neutrophil oxidative respiratory burst and an inflammatory response suggest that their pharmacological potential must await a more detailed analysis of these diverse roles [96,108-112].

### Short interfering RNA

In all metazoan cells, double-stranded RNA (dsRNA) triggers a cascade of biochemical reactions, collectively named RNA interference (RNAi). The net result of RNAi is to silence or degrade any RNA that is complementary to either strand of the dsRNA. If the target is mRNA, the net result is abrogation of the corresponding protein synthesis, resulting in 'knockdown' of the gene (as opposed to 'knockout', in which the DNA gene itself is deleted). Of relevance to research scientists and clinicians, the RNAi response can be triggered by synthetic dsRNA 18 to 22 base-pairs long, called siRNA in both cell culture [91,113] and in animals [114]. Historically, the first study demonstrating the antiviral use of appropriately designed synthetic siRNA targeted another respiratory virus, namely respiratory syncytial virus, a lower respiratory tract pathogen of paramount importance in pediatrics [113]. Anti-influenza siRNAs followed soon after [90]. For both viruses, intranasally administered siRNA was promptly delivered to the lungs and showed significant efficacy and protection of animals [115-118]. For respiratory syncytial virus, an inhaler-based application was also found to be useful in the mouse model, which should work for influenza as well [114,117].

The siRNAs in general enjoy several advantages over organic chemical drugs (such as Tamiflu and Relenza) [117]. First, the siRNA 'drugs' can be rapidly synthesized and scaled up for production. Second, in the event of viral resistance to one siRNA, a different siRNA targeting another viral sequence can be used. Third, regardless of sequence, all siRNAs use the same synthetic chemistry and hence the same manufacturing process. Finally, unlike many pharmacologically active organic compounds, siRNAs are water-soluble. Nonetheless, a clinically viable anti-influenza siRNA must meet a number of criteria, including specific tissue delivery (lung and the adjoining airspace), low toxicity and immune reaction, and pharmacokinetic stability.

A number of siRNA sequences, targeting various genes of influenza virus (Figure 3), have been tested over the last few years [90,115,118-120]. Recently, with better knowledge of siRNA design parameters and availability of appropriate bioinformatic algorithms, a more comprehensive siRNA repertoire covering a larger number of influenza viral genes in a variety of strains and isolates has been published [121-123]. Although no siRNA is yet commercially available for influenza treatment, Siranomics, Inc. (Gaithersburg, MD, USA) is developing the proprietary STP702 (FluQuit), a cocktail of siRNA designed to inhibit conserved regions in H1N1 and H5N1 strains of the influenza virus [124]. The ultimate goal would be to develop STP702 with demonstrated activity against multiple influenza A strains including H1N1, H5N1, H3N2, H7N2 and H9N2 [124].

### Drugs targeting the 'host interactome' of influenza

The limited number of influenza genes that can be targeted and the problems of resistance have made the targeting of host genes that are necessary for virus growth, nicknamed host interactome [125], an attractive new paradigm. It is also built on the premise that short-term inhibition of these host functions to treat an acute infection would not have major side effects. The concept of pharmacologically relevant genes, commonly called druggable genes, already exists. For example, essentially all successful cancer chemotherapeutic drugs target host functions. The anti-HIV drug, maraviroc, targets the viral co-receptor C-C chemokine receptor 5, and thereby prevents vial entry [126].

In the past few years, a number of comprehensive, genome-wide studies have identified host genes essential for virus growth, primarily through the use of siRNA libraries against the host genome and innovative, high-throughput reporter viral assays. In influenza, at least five such studies were conducted that used diverse readouts in different cell types, multiple virus strains and siRNA libraries targeting about 22,000 host genes [127-131]. Each screen identified a few hundred hits; intriguingly, however, not one hit was common to all five screens, perhaps underscoring the differences in their methodology and assay variables. Nonetheless, analysis of the hits revealed genes common to subsets of screens; for example, 85 genes were common to two or more of the screens, 72 genes were common to two of the five screens, 8 were common to three screens, and 5 were common to four screens [125]. These five genes code for: archain 1, ATPase, H^+ ^transporting, lysosomal accessory protein 1, coatamer protein complex, α subunit, coatamer protein complex, γ subunit, and nuclear RNA export factor 1. The functional categories, over-represented in the 85 cellular genes mentioned above, include ribosomal proteins, COPI (coat protein) vesicles, ATPase complex, spliceosomal proteins, nuclear envelope and kinase/signaling proteins, which underscores the many areas of the host that the virus co-opts [125,132]. Of these, nearly 50 are considered druggable, according to the Integrated Druggable Genome Database available from Sophic (http://www.sophicalliance.com/) [125,133], many of which can be pursued as targets of anti-influenza drug discovery. A few leads have been already confirmed by gene-specific analysis [125,134,135]. For example, an inhibitor (KN93; Figure 2) of the Ca^+2^/calmodulin-dependent kinase (CAMK2B), a gene identified in one genome-wide screen [131], inhibited influenza virus replication. Similarly, an inhibitor (TG003) of the CD-like kinase 1 that was found from another screen [130] also inhibited influenza virus growth. The third example is of p27, also found in the same screen [130]; the p27 knockout mice were found to be not only viable but also substantially resistant to virus growth, suggesting that p27 is a viable drug target.

In a separate recent screen, host genes specifically important for influenza viral polymerase (RdRP) function were identified, which are also potential candidates for antiviral drug development [136]. A chemical biology screen of 200,000 synthetic compounds recently identified naphthalimides as an antiviral chemical class that activated a new host defense factor, REDD1, which in turn inhibited influenza NS1 and viral replication [137]. Thus, activation of REDD1, rather than inhibition, can be developed as a new anti-influenza regimen.

## Conclusion

The current influenza treatments (Tamiflu, Relenza) target the viral NA and are quite effective. However, history has taught us that the virus mutates rapidly and becomes resistant to antivirals, as exemplified by the discontinuation of the once-effective adamantanes, the viral M2 inhibitor family. Viral resistance against the NA inhibitors has in fact begun to emerge recently, and their continuous use may lead to wide-spread selection of such mutants, making the population vulnerable to a drug-resistant epidemic. It is clearly important to have new antivirals in our anti-influenza arsenal. Based on research efforts, there appears to be five promising new anti-influenza regimens. The first of these is new compounds screened against old and new viral targets, such as NA, HA, the N protein [138] and RdRP subunits, or even the M2 ion channel [139]. Recall that NS1 is a major anti-immune function of the virus, and drug development against it has recently begun [140], with the identification of one inhibitory compound, NSC125044 (Figure 2) that reduced virus growth to virtually the same extent as an NS1-deleted virus. Clearly, these studies are promising, and need to be expanded. The second possible regime comprises siRNA, provided that the recognized hurdles of siRNA delivery, stability and specificity are resolved to a clinically acceptable level [141,142]. Third are new treatments that target any of the recently identified druggable host factors, essential for virus replication. Fourth, multiple drugs cocktail, targeting two viral functions [143] or one viral and one cellular function can be developed, the latter including inflammatory players (for example, NF-κB, sphingosine, chemokines) commonly activated in influenza. Finally the fifth possibility for new anti-influenza regimens is naturally occurring innate immune peptides, such as defensins and cathelicidins, that can be further optimized for a proper balance between their anti-influenza and signaling effects.

## Abbreviations

dsRNA: double-stranded RNA; HA: hemagglutinin; HNP: human neutrophil peptide; M2: matrix protein 2; NA: neuraminidase; NF-κB: nuclear factor kappa B; NP: nucleocapsid protein; NS: nonstructural; RdRP: RNA-dependent RNA polymerase; RNAi: RNA interference; siRNA: short (or small) interfering RNA.

## Competing interests

The author is a Scientific Founder of Sirnaomics, Inc.

## Author information

The author is a Professor at the Department of Biological, Geological and Environmental Sciences and is also Director of the Center for Gene Regulation in Health and Disease at the Cleveland State University. He holds adjunct appointment in the Department of Molecular Genetics at the Cleveland Clinic, Cleveland, Ohio.

## Pre-publication history

The pre-publication history for this paper can be accessed here:

http://www.biomedcentral.com/1741-7015/10/104/prepub
